# Long-term effects of early antiretroviral initiation on HIV reservoir markers: a longitudinal analysis of the MERLIN clinical study

**DOI:** 10.1016/s2666-5247(21)00010-0

**Published:** 2021-03-23

**Authors:** Marta Massanella, Rachel A Bender Ignacio, Javier R Lama, Amélie Pagliuzza, Sayan Dasgupta, Ricardo Alfaro, Jessica Rios, Carmela Ganoza, Delia Pinto-Santini, Trupti Gilada, Ann Duerr, Nicolas Chomont

**Affiliations:** **Centre de Recherche du CHUM, Montreal, QC, Canada** (M Massanella PhD, N Chomont PhD); **Département de Microbiologie, Infectiologie et Immunologie, Université de Montréal, Montreal, QC, Canada** (M Massanella, A Pagliuzza MSc, N Chomont); **Fred Hutchinson Cancer Research Center, Seattle, WA, USA** (R A Bender Ignacio MD, S Dasgupta PhD, D Pinto-Santini PhD, T Gilada MD, Prof A Duerr MD); **University of Washington, Seattle, WA, USA** (R A B Ignacio, Prof A Duerr); **Asociación Civil Impacta Salud y Educación, Lima, Perú** (J R Lama MD, R Alfaro MS, J Rios MS, C Ganoza MD)

## Abstract

**Background:**

Early antiretroviral therapy (ART) initiation (ie, within 3 months of infection) limits establishment of the HIV reservoir. However, the effect of early ART initiation on the long-term dynamics of the pool of infected cells remains unclear.

**Methods:**

In this longitudinal analysis, we included cisgender men who have sex with men (MSM) and transgender women (aged 18–54 years) at high risk for HIV infection, enrolled in the ongoing longitudinal MERLIN study in Peru between Oct 28, 2014, and Nov 8, 2018. Participants were eligible if they had been infected with HIV less than 90 days before enrolment, and if they had cryopreserved peripheral blood mononuclear cell (PBMC) samples. Participants were stratified into three groups on the basis of whether they initiated ART at 30 days or less (acute group), at 31–90 days (early group), or more than 24 weeks (deferred group) after the estimated date of detectable infection. PBMC samples were collected before ART initiation and longitudinally for up to 4 years on ART. The main outcomes were to establish the size of the HIV reservoir before ART initiation and to assess the effect of the timing of ART initiation on the decay of the HIV reservoir over 4 years follow-up. We quantified viral load, and isolated CD4 cells to quantify total HIV DNA, integrated HIV DNA and 2-long terminal repeat circles. Longitudinal analysis of active and inducible HIV reservoirs were measured by quantifying the frequency of CD4 cells producing multiply-spliced HIV RNA ex vivo and after in-vitro stimulation with a tat/rev induced limiting dilution assay (TILDA). A mixed-effects model from the time of ART initiation was used to measure longitudinal decays in viral loads and each HIV reservoir measure in each of the three groups.

**Findings:**

We included 56 participants in this analysis, all of whom were MSM: 15 were in the acute group, 19 were in the early group, and 22 were in the deferred group. Participants in all three groups had similar levels of all HIV reservoir markers before ART initiation. All participants, including those in the acute group, had a pool of transcriptionally silent HIV-infected cells before ART initiation, as indicated by a substantial increase in TILDA measures upon stimulation. Longitudinal analysis over 4 years of ART revealed a biphasic decay of all HIV persistence markers, with a rapid initial decline followed by a slower decay in all participants. During the first-phase decay, the half-lives of both total and integrated HIV DNA and TILDA measures were significantly shorter in the acute group than in the early and deferred groups. During the second-phase decay, HIV reservoir markers continued to decline only in participants in the acute group.

**Interpretation:**

Participants who initiated ART within 30 days or less of HIV infection showed a steeper and more sustained decay in HIV reservoir measures, suggesting long-term benefit of acute ART initiation on reservoir clearance.

**Funding:**

The US National Institutes of Health and the Canadian Institutes for Health Research.

## Introduction

Antiretroviral therapy (ART) suppresses HIV replication to undetectable levels in plasma. However, a small pool of long-lived, infected memory CD4 cells is unavoidably established during the first few weeks of HIV infection^1,2^ and remains the major obstacle to HIV remission.^3–5^

During acute HIV infection (ie, ≤30 days), plasma viral load increases rapidly, peaking after 3–4 weeks before declining to a viral set point.^6–8^ Initiating ART early (ie, within 3 months of infection) leads to a rapid decay in viraemia^9^ and drastically limits the size of the HIV reservoir.^10–19^ Nonetheless, early ART initiation does not prevent establishment of the reservoir, as indicated by the occurrence of viral rebound when treatment is interrupted, even in individuals who have initiated treatment at the earliest stages of HIV infection.^20,21^ Studies in non-human primates have revealed that a long-lived, replication-competent HIV reservoir can already be established in the first few days of Simian immunodeficiency virus infection, when plasma viraemia is not yet detectable.^22,23^ Although early ART initiation does not prevent establishment of the HIV reservoir, several observational studies have suggested that prompt ART initiation (ie, within 1–6 months of infection) might be associated with a more rapid decay in the pool of infected cells compared with delayed ART initiation (ie, >6 months after infection),^10–16,19^ which could accelerate reservoir clearance when combined with other eradication strategies. Several groups have described a biphasic decay of the HIV DNA reservoir on ART initiation, with a rapid initial decline followed by a second slower decay.^13,24–26^ Even though HIV DNA dynamics follow a similar two-phase decay pattern in early or chronically treated individuals, early ART initiation could be associated with a more rapid initial clearance of the HIV reservoir and a slower but continuous decay after long-term therapy compared with delayed ART initiation.^13,16,19,27^ Indeed, one study, published in 2020, in which an intact proviral DNA assay was used, revealed that intact proviruses might decay more rapidly than defective proviruses.^28^ However, the size of the inducible reservoir after long-term therapy has not yet been assessed in a large longitudinal study. Some studies have used the quantitative viral outgrowth assay to evaluate the dynamics of the functional HIV reservoir in a small number of participants.^10,12^ However this assay largely underestimates the size of the inducible HIV reservoir and has a narrow dynamic range. In contrast, assays that measure the frequency of CD4 cells with inducible RNAs, such as the tat/rev induced limiting dilution assay (TILDA),^29^ provide more sensitivity and a greater dynamic range for monitoring reductions in the HIV reservoir over time.

The aim of this study was to assess the effects of the timing of ART initiation on the longitudinal persistence of HIV DNA and reservoir markers over 4 years of ART.

## Methods

### Study design and participants

In this longitudinal analysis, we included cisgender men who have sex with men (MSM) and transgender women (aged 18–54 years) at high risk for HIV infection, enrolled in the ongoing longitudinal MERLIN study (NCT02744040) in Peru between Oct 28, 2014, and Nov 8, 2018.

The MERLIN study follows a subset of participants in the Sabes study (NCT01815580),^30^ which enrolled 2109 cisgender MSM and transgender women (aged 18–73 years) at high risk for HIV infection in Lima, Peru, between Aug 5, 2013, and Sept 29, 2015. Participants were screened monthly for HIV RNA and serology to detect incident infections. HIV screening was done on whole blood using the third-generation HIV antibody test, Alere Determine HIV-1/2 (Alere, Waltham, MA, USA). Blood samples negative for HIV antibodies were pooled and tested for HIV RNA with a nucleic acid amplification test (Liat HIV Quant Assay [IQuum, Hercules, CA, USA). Positive pools were deconvoluted to identify HIV-positive samples using a fourth-generation HIV enzyme immune-assay (Bio-Rad Laboratories, Marlborough, MA, USA) or HIV RNA tests. The Abbott RealTime HIV-1 Viral Load Assay (Abbott Molecular, Plaines, IL, USA) was used to confirm HIV infection. Participants were eligible for the Sabes study if they were seronegative but positive for HIV RNA (acute infection) or were seropositive less than 90 days from their last negative HIV RNA or serological test (early infection). 216 individuals who were diagnosed during acute and early HIV infection consented to additional follow-up. Participants were enrolled within days of HIV diagnosis and randomised within these strata to receive ART either immediately or after 24 weeks deferral (or sooner if they met local ART initiation criteria).^31^ Enrolment of participants in the deferred group occurred before 2016, when Peru adopted the WHO “treat all” to end AIDS recommendations.^31,32^ The ongoing longitudinal study, MERLIN, follows a subset of Sabes participants on suppressive ART (defined as an undetectable viral load after 1 year on ART, no more than two consecutive blips of >100 HIV RNA copies per mL of plasma, and a maximum allowed viral blip of <500 HIV RNA copies per mL), with the aim of understanding how differential timing of ART initiation affects long-term outcomes (ie, the HIV reservoir).

In this analysis, participants were categorised by the interval between the estimated date of detectable infection (EDDI) and ART initiation (appendix p 4); an interval that included the time from HIV acquisition to the date of enrolment, randomisation, and actual ART initiation. EDDI was established by use of the published Consortium for the Evaluation and Performance of HIV Incidence Assays (CEPHIA) calculator, which considers all negative and positive HIV test results and their uncertainty intervals, as previously described.^31,33^ Contraindication for use of study antiretroviral drugs was an exclusion criterion. This analysis includes 56 participants (enrolled between Oct 28, 2014, and Nov 8, 2018) with cryopreserved peripheral blood mononuclear cells (PBMCs) available. We stratified participants into three groups on the basis of whether they initiated ART at 30 days or less (acute group), at 31–90 days (early group), or more than 24 weeks (deferred group) after the EDDI (appendix p 4). PBMCs were obtained before ART initiation and at weeks 1, 2, 4, 8, 16, 24, 96, and 192.

All participants provided written informed consent, and the study was approved by the ethics committee of the non-governmental organisation Impacta Salud y Educación, the Peruvian National Institute of Health, and the Institutional Review Boards of the Fred Hutchinson Cancer Research Center and the Centre Hospitalier de l’Université de Montréal.

### Procedures

To measure total and integrated HIV DNA and 2-long terminal repeat (LTR) circles, total CD4 cells were isolated from cryopreserved PBMCs by immunomagnetic negative magnetic selection (EasySep Human CD4+ T Cell Enrichment Kit; STEMCELL Technologies, Vancouver, BC, Canada). Pellets of enriched CD4 cells were digested with proteinase K; total (5′-LTR-*gag*) and integrated (3′-LTR-*alu*) HIV DNA, and the 2-LTR junction (2-LTR circles assay) were quantified by real-time PCR, as described previously.^34^ Samples in which fewer than 50 000 cells were analysed were excluded from the analysis.

HIV reservoirs were also measured by quantifying the frequency of CD4 cells producing multiply-spliced HIV RNA. When sufficient viable cells were available, the frequency of productively infected cells (for samples up to 8 weeks after ART initiation) were measured by TILDA ex vivo, and the size of the inducible HIV reservoir (at all timepoints) was measured by TILDA after in-vitro stimulation (appendix p 2).^29^ HIV reservoir analyses were done at different time points based on the availability of the samples (see appendix p 4).

### Outcomes

The main outcomes were to measure the size of the HIV reservoir before ART initiation and to assess the effect of the timing of ART initiation on the decay of HIV reservoir measures (including total and integrated HIV DNA, 2-LTR circles, and TILDA ex vivo or after in-vitro stimulation) over 4 years follow-up.

### Statistical analysis

HIV DNA and TILDA values were expressed as log_10_ ([HIV copies per 10⁶ CD4 cells] + 1). Results were represented as medians (IQR). To compare values between the acute, early, and deferred groups before ART initiation we used the Kruskal-Wallis test for continuous variables and the Freeman-Halton extension to Fisher’s exact test for categorical variables. The Wilcoxon matched-pairs signed rank test was used to compare values in the deferred group at enrolment and at 24 weeks thereafter (ART start date) for continuous variables and Fisher’s exact test for categorical variables. Correlations between continuous variables were assessed with Spearman’s rank correlation coefficient. The longitudinal decays in viral loads and each HIV reservoir measure were analysed by use of a mixed-effects model from the time of ART initiation up to 196 weeks in each of the three groups (appendix pp 2–3). The significance level was set at 0·05, and multiple hypothesis testing was accounted for with the Tukey’s honest significant difference test.^35^

Statistical analyses were done in R software, version 3.5.1, and Prism 7 software.

### Role of the funding source

The funders of the study had no involvement in study design, data collection, data analysis, data interpretation, or writing of the report, nor in the decision to submit the paper for publication.

## Results

We included 56 participants in this analysis, of whom 15 were in the acute group, 19 were in the early group and 22 were in the deferred group. All participants were MSM with a median age of 27 years (IQR 22–30). Additional characteristics of the participants at enrolment are shown in table 1 and in the appendix (p 4).

**Figure 1:**
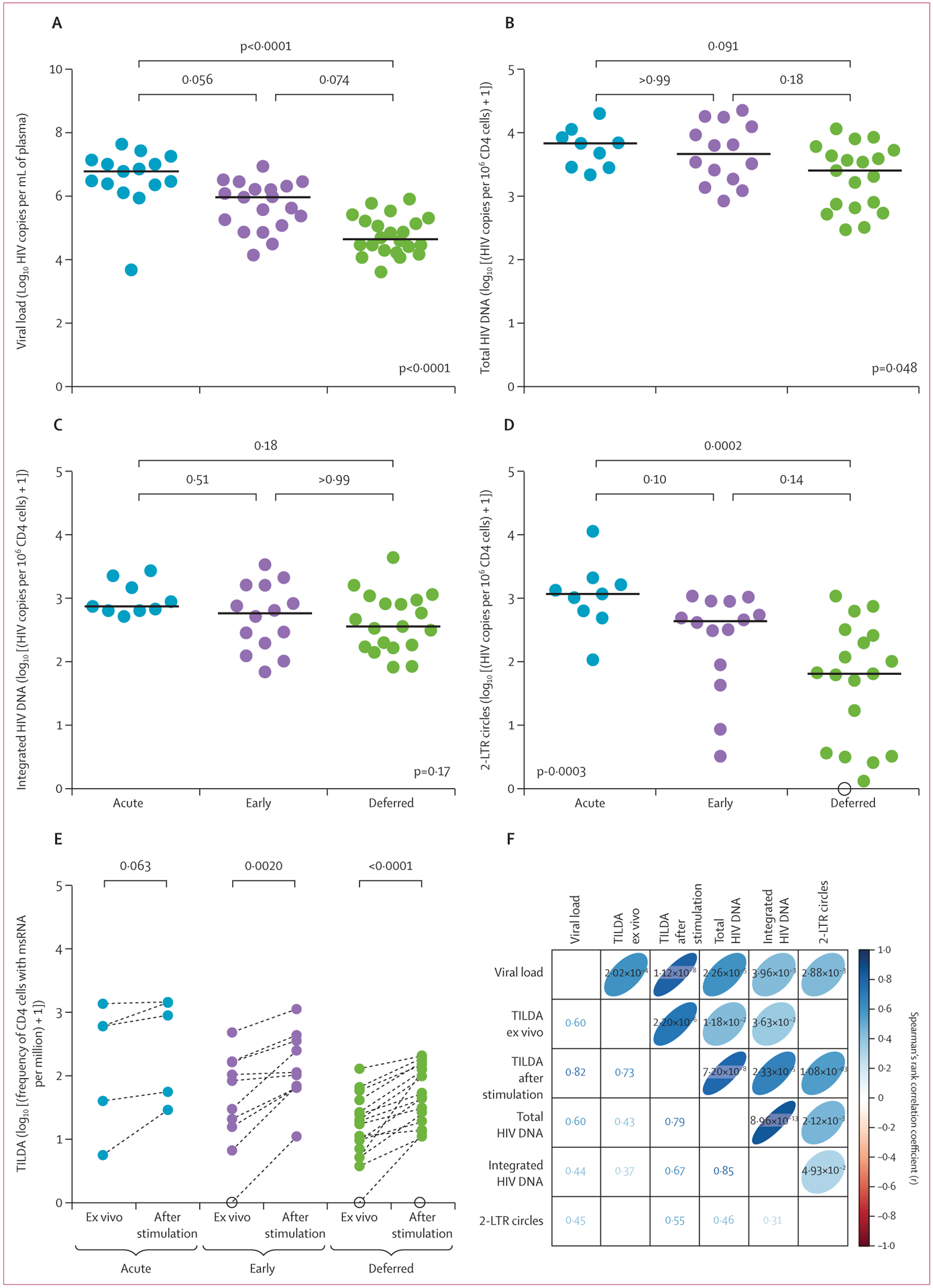
Virological markers of HIV persistence before ART initiation. Plasma viral load (A), frequency of CD4 cells harbouring total HIV DNA (B), integrated HIV DNA (C), and 2-LTR circles (D), and frequency of CD4 cells producing msRNA spontaneously (TILDA ex vivo) or after 12 h stimulation (E) in participants stratified by whether they received ART within 30 days or less (acute group; n=9), at 31–90 days (early group; n=14), or more than 24 weeks (deferred group; n=19) after the EDDI. (F) Correlation matrix between the virological markers of HIV persistence. In A–E, samples with undetectable levels of virological markers (ie, less than the lower limit of the detection of the assay, which can vary according to the number of cells analysed), were considered as 0 for statistical analyses and are indicated as open circles. In A–D, p values were derived from the Kruskal-Wallis test, and in E, p values were derived from the Wilcoxon test for paired samples. In F, *r* values from Spearman correlation test are indicated in the bottom left part of the matrix and are reflected by the colour of the ellipses in the top right (blue indicates a positive correlation and red indicates a negative correlation); the shape of the ellipsis reflects the level of significance (ie, a narrower ellipsis reflects a lower p value), which are also included in the upper part of the matrix. Only significant correlations are shown in F. ART=antiretroviral therapy. TILDA=tat/rev induced limiting dilution assay. msRNA=multiply-spliced RNA. 2-LTR=2-long terminal repeat. EDDI=estimated date of detectable infection.

To assess the seeding of the HIV reservoir, we measured total and integrated HIV DNA and 2-LTR circles, as well as the active and inducible reservoirs in CD4 cells from all participants before ART initiation. As expected, participants in the acute group had significantly higher viral loads than those in the early (p=0·056) and deferred groups (p=0·0074; figure 1A). Similarly, total and integrated HIV DNA levels in the acute group were higher than in the deferred group, but these differences were not significantly different (p=0·091 for total HIV DNA and p=0·18 for integrated HIV DNA; figure 1B, C). These results suggest that a large pool of HIV-infected cells was rapidly established during the first 30 days of HIV infection and remained relatively stable in size. We also measured the frequency of cells harbouring 2-LTR circles as a proxy of active HIV replication. Similar to viral load, participants in the acute group showed significantly higher frequencies of 2-LTR circles compared with those in the deferred group (p=0·0002; figure 1D). Furthermore, the frequency of cells expressing multiply-spliced RNA significantly increased on in-vitro stimulation relative to ex-vivo condition in most participants in the acute (five of five participants, p=0·063), early (ten of ten participants, p=0·0020), and deferred (16 of 17 participants, p<0·0001) groups, indicating that a pool of latently infected cells had already been established in most participants (figure 1E). Importantly, all HIV reservoir measures were significantly associated with each other and with plasma viral load (figure 1F), suggesting that plasma viraemia could be used as a surrogate marker of the size of the initial pool of infected cells before ART initiation.

To confirm these cross-sectional observations, we analysed matched longitudinal samples collected from participants in the deferred group at enrolment and at week 24 (before ART initiation; appendix p 7). As expected, viral loads decreased significantly during the first 24 weeks after enrolment (p<0·0001; appendix p 5). Similarly, the frequency of cells harbouring total HIV DNA (p=0·0003) and 2-LTR circles (p=0·016) decreased between enrolment and week 4, whereas the levels of integrated HIV DNA remained stable over this period (p=0·37). In these matched samples from untreated participants, stimulation of CD4 cells with phorbol 12-myristate 13-acetate and ionomycin increased the frequency of cells producing multiply-spliced RNA (appendix p 5), indicating that a pool of latently infected cells was already established during early HIV infection and was maintained for 24 weeks.

**Figure 2:**
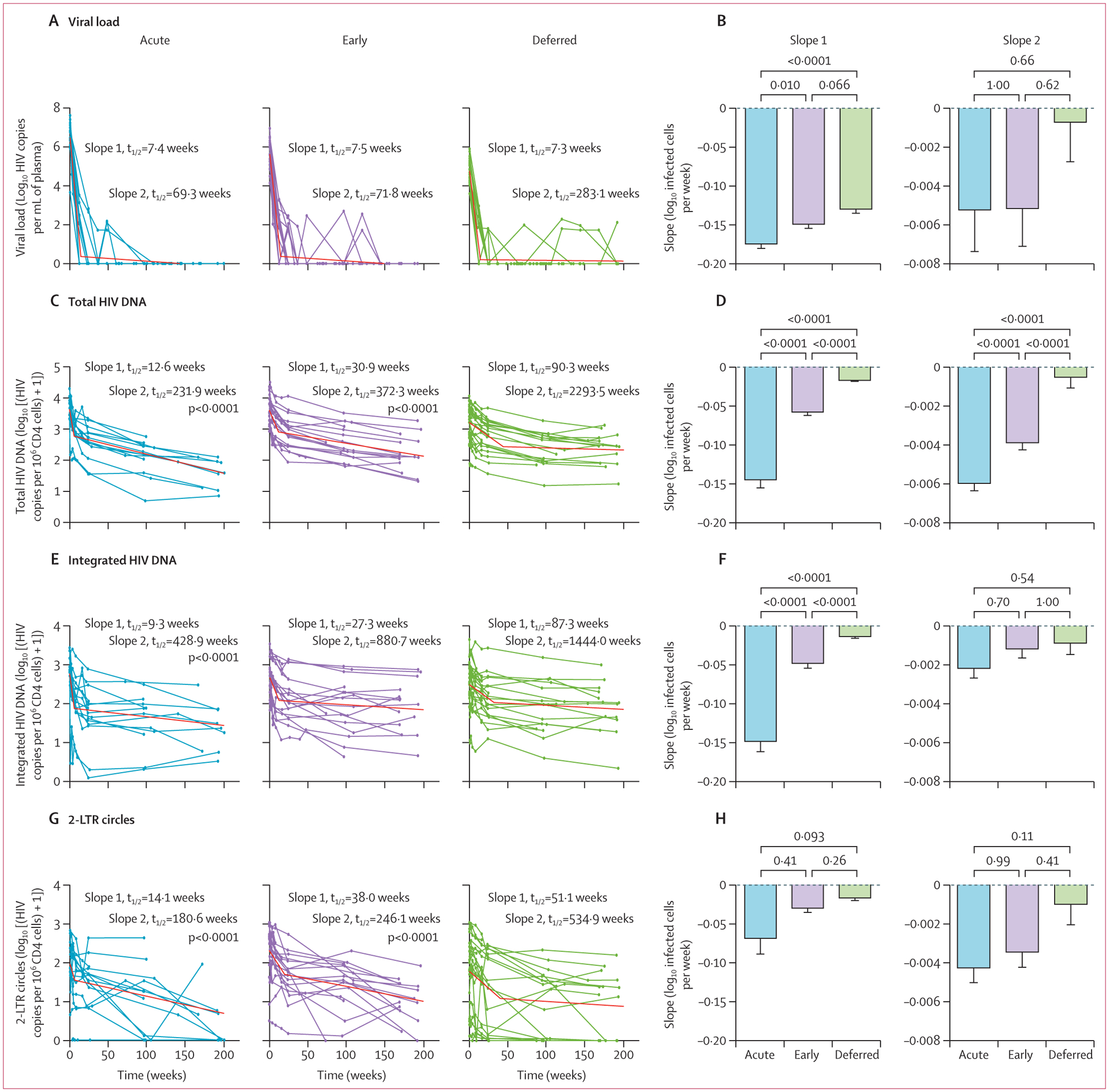
Longitudinal analysis of viral load and HIV DNA decay during up to 4 years on ART Longitudinal analysis of plasma viral loads (A, B), total HIV DNA (C, D), integrated HIV DNA (E, F), and 2-LTR circles (G, H) in enriched CD4 cells from participants stratified by whether they received ART within 30 days or less (acute group; n=15), at 31–90 days (early group; n=19), or more than 24 weeks (deferred group; n=22) after the estimated date of detectable infection. Samples collected immediately before ART initiation (0 weeks) were included. Participants were followed for up to 4 years on ART. For all decay slopes (A, C, E, and G), a two-phase segmentation model of viral load and HIV DNA was applied; for each group and each virological marker, the optimal change point was selected by use of the minimisation of the Akaike information criterion. Each dot represents a timepoint analysed for a given participant, and samples from the same individual are connected. The best fitted model for each virological marker is presented in red. The p values of slope 2 reflect a significant decay of a given marker. The t_1/2_ values of slopes 1 and 2 are indicated. In B, D, F, and H, comparisons of slopes 1 and 2 for each group and virological marker calculated from the models are shown; error bars indicate the SEs. ART=antiretroviral therapy. t_1/2_=half-life. 2-LTR=2-long terminal repeat.

We then assessed the effect of the timing of ART initiation on the decay of HIV reservoir measures over time. Plasma viraemia fitted to a two-phase decay model in all groups (table 2; figure 2A). There was a significantly faster first-phase decay of plasma viraemia in the acute group compared with the early (p=0·010) and deferred (p<0·0001) groups, whereas no difference in the rate of first-phase decay was observed between the early and deferred groups (table 2; figure 2B). The rate of second-phase decay in plasma viraemia did not differ significantly among the three study groups.

Similar to plasma viraemia, total and integrated HIV DNA levels fitted to a two-phase decay model (table 2; figure 2C, E). Despite similar estimated intercept values in each group, the acute group showed significantly faster initial decays of both total and integrated DNA compared with the other groups (p<0·0001 for all comparisons; table 2; figure 2D, F). In the acute group, the initial half-life of total HIV DNA was 12·6 weeks and of integrated HIV DNA was 9·3 weeks (figure 2C, E). The early group also showed a significantly faster rate of decay of total HIV DNA (half-life 30·9 weeks in the early group *vs* 90·3 weeks in the deferred group; p<0·0001) and integrated HIV DNA (27·3 weeks *vs* 87·3 weeks; p<0·0001) compared with the deferred group.

During the second-phase decay, total HIV DNA levels continued to decrease in the acute (half-life 231·9 weeks; slope statistically different from 0 p<0·0001) and early groups (372·3 weeks; p<0·0001), whereas total HIV DNA levels remained stable in the deferred group (2293·5 weeks; p=0·954; table 2; figure 2C). Similar to the first-phase decay, the second-phase decay of total HIV DNA was more pronounced in the acute group compared with the early (p<0·0001) and deferred groups (p<0·0001). In addition, the early group displayed a significantly faster rate of decay of total HIV DNA compared with the deferred group (p<0·0001). In contrast to total HIV DNA, no differences were observed in the rate of second-phase decay of integrated HIV DNA among the three groups (figure 2E, F). However, a continuous slower decay was observed in the acute group only (slope statistically different from 0 p<0·0001; table 2).

Although the frequency of cells harbouring 2-LTR circles also followed a two-phase decay model for all groups, no significant differences in the first-phase and second-phase decay slopes were observed among the three groups (table 2; figure 2G, H). Notably, the frequency of 2-LTR circles continued to decrease significantly during the second-phase decay in the acute (p<0·0001) and early (p<0·0001) groups. These results indicate that, although 2-LTR circles can still be detected after years on ART, they are more labile than total and integrated forms of HIV DNA.

**Figure 3:**
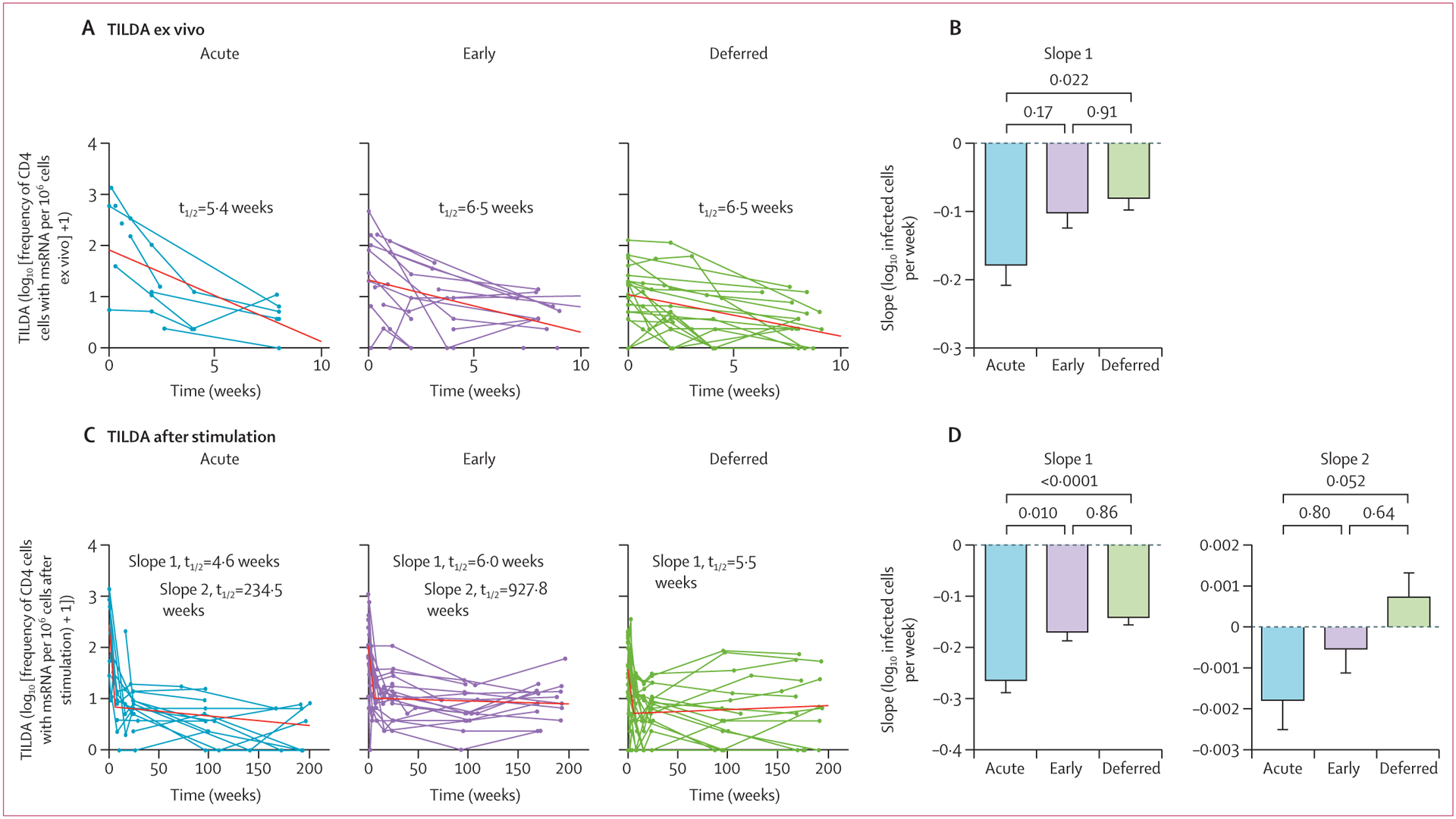
Longitudinal analysis of active (TILDA ex vivo) and inducible (TILDA after stimulation) HIV reservoirs during up to 4 years on ART. Longitudinal analysis of TILDA ex vivo (A, B) and after stimulation (C, D) measures in enriched CD4 cells from participants stratified by whether they received ART within 30 days or less (acute group; n=15), at 31–90 days (early group; n=19), or more than 24 weeks (deferred group; n=22) after the estimated date of detectable infection. Samples collected immediately before ART initiation (0 weeks) were included. Participants were followed for up to 4 years on ART. We applied a linear function for TILDA ex-vivo measures. We applied a two-phase segmentation model of TILDA after stimulation measures; for each group, the optimal change point was selected by use of the minimisation of the Akaike information criterion. For all decay slopes (A and C), each dot represents a timepoint analysed for a given participant, and samples from the same individual are connected; the best fitted model for each virological marker is represented in red. The t_1/2_ values of slopes 1 and 2 are indicated. Comparisons of slope 1 in B, and slopes 1 and 2 in D for each group and virological marker calculated from the models are shown; error bars indicate SEs. TILDA=tat/rev induced limiting dilution assay. ART=antiretroviral therapy. msRNA=multiply-spliced mRNA. t_1/2_=half-life.

We evaluated the decay in the frequency of productively infected cells measured by TILDA ex vivo during the first 8 weeks of ART, which fitted a one-phase decay model (figure 3A). The acute group showed a faster rate of decay of productively infected cells than the deferred group (p=0·022; table 3; figure 3B), which is likely to reflect the fact that the initial frequencies of cells expressing tat/rev transcripts at baseline were higher in this group compared with the early and deferred groups.

We then evaluated the decay in the frequency of CD4 cells harbouring inducible HIV proviruses over 4 years of ART using TILDA after stimulation measures. The data fitted a two-phase decay model (table 3; figure 3C). The acute group showed a significantly faster rate of first-phase decay of the inducible reservoir compared with the early (p=0·010) and deferred (p<0·0001) groups (figure 3D), although the differences in half-lives among the acute (4·6 weeks), early (6·0 weeks), and deferred (5·5 weeks) groups were small. During the second-phase decay, the acute group displayed a faster rate of decay than the deferred group (p=0·052; table 3; figure 3D).

Total and integrated HIV DNA levels, and TILDA measurements were significantly associated with each other after 24, 96, and 192 weeks of ART (appendix p 6). In addition, levels of HIV reservoir markers measured before ART initiation and after 24, 96, and 192 weeks of ART were significantly associated, indicating that the size of the initial pool of infected cells predicts the size of the reservoir, even after long-term therapy.

## Discussion

Several studies have shown that initiating ART during acute HIV infection drastically limits the size of the HIV reservoir.^10–16,19^ However, early diagnosis is still an overwhelming challenge in a real-world context, whereby frequent testing in high-incidence populations remains rare.^36^ In this study, we compared the size of the HIV reservoir in individuals initiating ART during acute (≤30 days), early (31–90 days), and early chronic (6–9 months) infection, to establish whether the faster and sustained reduction of HIV reservoirs in individuals starting ART during the first month of infection extend to those initiating therapy after this point. To our knowledge, this is the first study of immediate versus deferred ART that evaluated the effect of the timing of ART initiation on HIV reservoirs in a longitudinal setting.

We found that a large pool of HIV-infected cells was rapidly established during the first 30 days of HIV infection and remained stable, as indicated by similar baseline levels of HIV reservoir markers among the three groups. This observation is consistent with previous studies showing that the levels of total and integrated HIV DNA increase rapidly during the first few weeks of infection.^11,14,19,37–39^ Of note, there was a significant difference in the frequency of cells harbouring 2-LTR circles among the three groups before initiation of ART (p=0·0003), which is likely to reflect different levels of HIV replication.^40–42^

Several studies have reported a rapid initial decay in the levels of total HIV DNA on ART initiation, followed by a slower decay after several weeks of therapy.^13,15,24–26,39^ Our study corroborates and extends these observations, as we observed a rapid decay in total and integrated HIV DNA, and in the frequency of cells with inducible HIV RNA during the first weeks of ART. This rapid initial decay was followed by a slower decay, probably reflecting heterogeneous populations of infected cells characterised by different clearance rates.^43^ Of note, we found that different markers of HIV persistence displayed different rates of decay during the first weeks of ART. The inducible reservoir measured by TILDA after stimulation showed a steeper decline than HIV DNA measures, possibly reflecting the preferential clearance of productively infected cells over those containing latent proviruses or viral genomes containing large defects. The faster rate of decay in reservoir markers in the acute group could have resulted from a stronger cytotoxic T lymphocyte pressure caused as a result of their preserved functions.^44^ This faster rate of clearance could also be due to the lower proportion of cytotoxic T lymphocyte escape mutations in the reservoir of these participants compared with those treated later;^45^ although, this factor remains controversial.^46^ As reported in our study, levels of integrated HIV DNA declined less rapidly than levels of total HIV DNA and 2-LTR circles,^14,19,47^ indicating that unintegrated HIV genomes are more labile, possibly because they are diluted by T-cell proliferation.

Initiating ART within 30 days or less of infection induced a faster initial decay of all HIV reservoir markers compared with initiating ART at 31–90 days or after 24 weeks of infection. These results are consistent with those of other studies,^13–15^ and indicate that early HIV reservoir markers are less stable when ART is initiated within the first month of infection. This reduced stability could be attributed to the rapid clearance of short-lived CD4 effector-memory T cells, with accelerated intrinsic decay kinetics, that might be the preferential target for HIV during acute infection.^14,19,48^ We and others^14,19^ previously reported that the long-lived CD4 central memory T cells are protected when ART is initiated early relative to other subsets, whereas these cells constitute a large part of the HIV reservoir in people initiating ART during chronic infection (ie, >6 months). Since CD4 central memory T cells are not only long-lived, but also display exquisite proliferative capacities that can replenish the CD4 effector memory T-cell reservoir when stimulated,^49^ their infection might contribute to the slower decay in reservoir markers in people who delay ART initiation. Additional studies are warranted to establish whether the type of cells in which HIV persists could explain the differential decay rates between these different groups.

Of note, the benefit of early ART was not limited to those initiating treatment during the first month of infection, since the first-phase decay of total and integrated HIV DNA was faster in individuals who initiated ART at 31–90 days after infection compared with those who initiated ART after 24 weeks. In contrast to our findings, Strain and colleagues^10^ did not observe a difference in the half-life of total HIV DNA between individuals who initiated ART before or after seroconversion (all within 6 months from the EDDI). In this previous study, decay of HIV DNA was evaluated in bulk PBMCs, whereas enriched CD4 cells were used in our analyses, which suggests that the apparent differences between studies could be attributed to CD4 cell recovery after ART initiation. Additionally, differences in ART regimens between studies could affect viral decay, and thereby contribute to these discrepancies.

We used TILDA measures to assess the transcriptionally competent and inducible reservoir as a surrogate for the quantitative viral outgrowth assay.^29^ In agreement with several studies showing a rapid decay in the size of the replication-competent reservoir, measured by the quantitative viral outgrowth assay,^10,50^ we observed a rapid initial decay in TILDA measures, which was faster in the acute group compared with the early and deferred groups. This observation contrasts that of a previous study, which used a quantitative viral outgrowth assay to measure the HIV reservoir, showing that the decay rates in infectious units per million cells were similar in acutely and chronically treated participants.^12^ Several factors could explain this difference. First, TILDA does not measure replication-competent HIV and is likely to overestimate the actual size of the viral reservoir. Second, we used total CD4 cells (as opposed to resting CD4 cells) in our analysis, thereby including activated CD4 cells, which can have a faster rate of decay than resting cells.

Since our study did not include individuals with chronic infection, we cannot comment on the potential beneficial effects of ART initiation during early chronic infection (ie, in participants in the deferred group) compared with infection of longer or unknown duration. Of note, other studies have not found a significant decrease in integrated HIV DNA levels in individuals starting ART during chronic infection,^14,17^ suggesting that initiation of therapy during the first 6–9 months of infection might still be beneficial.

The second-phase decay rates of all reservoir markers were slower than the first-phase decay rates and relatively similar across the three groups, suggesting that early ART primarily influences the slope of the first-phase decay.^14–16^ Nonetheless, and in accordance with previous studies,^13,26^ a measurable decay in integrated HIV DNA after long-term therapy was observed exclusively in participants initiating treatment within 30 days or less of infection. This observation indicates that acute ART initiation not only accelerates initial decay in the pool of infected cells, but also exerts long-term effects on the HIV reservoir. These effects could be attributed to the more labile nature of the infected cells in these individuals, in whom the long-lived CD4 central memory T cells are relatively protected by acute ART initiation.^14,19^ Although the mechanisms underlying the relative protection of CD4 central memory T cells during acute infection remain to be identified, one can hypothesise that the susceptibility of these cells increases after months of active HIV replication as a result of enhanced bystander T-cell activation. Alternatively, since antigen-driven proliferation contributes to the maintenance of the HIV reservoir,^49,51^ it is also possible that the reservoir in acutely treated participants is established in a small number of cells with a restricted number of antigenic specificities, whereas the repertoire of the reservoir in chronically infected participants is broader, and, therefore, more prone to undergo clonal expansions.

A limitation of our study is that we only included individuals assigned male sex at birth; therefore, we cannot exclude the possibility that the observed differences in the rate of decay of the reservoir might not be the same in cisgender women, in whom the reservoir has been shown to be less transcriptionally active.^52^

In conclusion, initiating ART within 30 days or less of infection is associated with a faster rate of decay in HIV reservoir markers compared with initiating therapy at 31–90 days or more than 24 weeks after infection. This notable benefit of the timing of ART initiation on the HIV reservoir suggests that the beneficial effects of early ART initiation might be more pronounced after long-term therapy. In addition, these observations suggest that promoting frequent HIV screening followed by prompt initiation of ART, particularly in individuals at a high risk of acquiring HIV, could provide opportunities to accelerate clearance of the HIV reservoir.

## Supplementary Material

1

## Figures and Tables

**Table 1:** Characteristics of participants on the day of ART initiation

	ART initiation			Intergroup comparisons			
	Acute (n=15)	Early (n=19)	Deferred (n=22)	Group-wise comparison p value	Acute *vs* early p value	Acute *vs* deferred p value	Early *vs* deferred p value
Male sex assigned at birth	15 (100%)	19 (100%)	22 (100%)	NS	NS	NS	NS
Age, years	28 (25–30)	27 (21–31)	25 (21–30)	NS	NS	NS	NS
Time from EDDI to enrolment, days	21 (18–23)	43 (36–57)	38 (27–57)	<0·0001	<0·0001	0·0002	NS
Time from EDDI to ART initiation, days	21 (18–23)	43 (36–57)	206 (196–220)	<0·0001	0·0076	<0·0001	0·0002
CD4 cell count, cells per μL	540 (296–598)	388 (229–506)	399 (315–565)	NS	NS	NS	NS
Percentage of CD4 cells	26% (20–37)	17% (11–23)	24% (17–18)	0·0053	0·0060	NS	0·054
CD8 cell count, cells per μL	775 (438–1119)	1072 (635–1717)	730 (566–920)	NS	NS	NS	NS
Percentage of CD8 cells	44 (31–54)	54 (48–72)	41 (38–48)	0·044	NS	NS	0·060
CD4/CD8 ratio	0·59 (0·41–1·01)	0·33 (0·16–0·49)	0·62 (0·34–0·68)	0·015	0·017	NS	0·084
ART regimen	··	··	··	NS	NS	NS	NS
Efavirenz, emtricitabine, and tenofovir disoproxil fumarate	10 (67%)	14 (74%)	16 (73%)	··	··	··	··
Elvitegravir, cobicistat, emtricitabine, and tenofovir disoproxil fumarate	3 (20%)	5 (26%)	6 (27%)	··	··	··	··
Elvitegravir, cobicistat, emtricitabine, and tenofovir alafenamide	2 (13%)	0	0	··	··	··	··
Viral load, log_10_ HIV copies per mL of plasma	6·8 (6·4–7·1)	5·96 (5·07–6·31)	4·64 (4·27–5·23)	<0·0001	0·056	<0·0001	0·0074
Total HIV DNA	9 (60%)	14 (74%)	19 (86%)	··	··	··	··
Log_10_ ([HIV copies per 10⁶ CD4 cells] + 1)	3·83 (3·45–3·99)	3·67 (3·24–4·13)	3·40 (2·81–3·72)	0·048	NS	0·091	NS
Integrated HIV DNA	9 (60%)	14 (74%)	19 (86%)	··	··	··	··
log_10_ ([HIV copies per 10⁶ CD4 cells] + 1)	2·87 (2·80–3·26)	2·76 (2·24–3·20)	2·55 (2·23–2·97)	NS	NS	NS	NS
2-LTR circles	9 (60%)	14 (74%)	19 (86%)	··	··	··	··
log_10_ ([HIV copies per 10⁶ CD4 cells] + 1)	3·07 (2·75–3·27)	2·64 (1·87–2·95)	1·81 (0·51–2·41)	0·0003	NS	0·0002	NS
TILDA ex vivo	5 (33%)	10 (53%)	18 (82%)	··	··	··	··
frequency of CD4 cells with msRNA per million cells	2·77 (1·17–2·95)	1·69 (1·10–2·21)	1·14 (0·71–1·47)	0·034	NS	0·055	NS
TILDA after stimulation	5 (33%)	10 (53%)	17 (77%)	··	··	··	··
frequency of CD4 cells with msRNA per million cells	2·94 (1·60–3·14)	2·02 (1·80–2·56)	1·66 (1·21–2·14)	0·057	NS	NS	NS

Data are n (%) or median (IQR), unless otherwise specified. Acute initiation of ART was within 30 days or less of the EDDI, early initiation was at 30–90 days of the EDDI, and delayed initiation was at more than 24 weeks after the EDDI. Groups were compared with the Kruskall-Wallis test. ART=antiretroviral therapy. NS=not significant. EDDI=estimated date of detectable infection. 2-LTR=2-long terminal repeat. TILDA=tat/rev induced limiting dilution assay. msRNA=multiply-spliced RNA.

**Table 2:** Two-phase decay models for viral load, total and integrated HIV DNA, and 2-LTR circles

	Group estimates			Intergroup comparisons		
	Acute initiation of ART	Early initiation of ART	Deferred initiation of ART	Acute *vs* early p value	Acute *vs* deferred p value	Early *vs* deferred p value
**Viral load** *						
Change point, weeks	14	14	14	··	··	··
Intercept	6·497 (6·153 to 6·840)	5·616 (5·308 to 5·925)	4·769 (4·476 to 5·061)	0·0023	<0·0001	0·0011
Slope 1	−0·438 (−0·466 to −0·410)	−0·375 (−0·400 to −0·350)	−0·326 (−0·351 to −0·301)	0·010	<0·0001	0·066
Slope 2	−0·003 (−0·005 to −0·001)	−0·003 (−0·004 to −0·001)	0·000 (−0·002 to 0·002)	NS	NS	NS
Slope 2 statistically different from 0 p value	NS	0·075	NS	··	··	··
Slope 1 *vs* slope 2 p value	<0·0001	<0·0001	<0·0001	··	··	··
**Total HIV DNA** †						
Change point, weeks	6	12	44	··	··	··
Intercept	3·642 (3·375 to 3·908)	3·599 (3·366 to 3·832)	3·211 (2·998 to 3·425)	NS	NS	NS
Slope 1	−0·145 (−0·164 to −0·126)	−0·058 (−0·067 to −0·050)	−0·018 (−0·021 to −0·015)	<0·0001	<0·0001	<0·0001
Slope 2	−0·006 (−0·007 to −0·005)	−0·004 (−0·005 to −0·003)	−0·001 (−0·021 to −0·015)	<0·0001	<0·0001	<0·0001
Slope 2 statistically different from 0 p value	<0·0001	<0·0001	NS	··	··	··
Slope 1 *vs* slope 2 p value	<0·0001	<0·0001	<0·0001	··	··	··
**Integrated HIV DNA** †						
Change point, weeks	6	12	32	··	··	··
Intercept	2·769 (2·465 to 3·074)	2·666 (2·402 to 2·931)	2·485 (2·243 to 2·728)	NS	NS	NS
Slope 1	0·149 (−0·174 to −0·124)	−0·049 (−0·06 to −0·038)	−0·014 (−0·019 to −0·01)	<0·0001	<0·0001	<0·0001
Slope 2	−0·002 (−0·003 to −0·001)	−0·001 (−0·002 to 0·000)	−0·001 (−0·002 to 0·000)	NS	NS	NS
Slope 2 statistically different from 0 p value	<0·0001	NS	NS	··	··	··
Slope 1 *vs* slope 2 p value	<0·0001	<0·0001	<0·0001	··	··	··
**2-LTR circles** †						
Change point, weeks	6	20	40	··	··	··
Intercept	1·961 (1·570 to 2·351)	2·317 (1·987 to 2·647)	1·783 (1·480 to 2·087)	NS	NS	NS
Slope 1	−0·069 (−0·108 to −0·031)	−0·031 (−0·041 to −0·020)	−0·017 (−0·024 to −0·011)	NS	0·093	NS
Slope 2	−0·004 (−0·006 to −0·003)	−0·003 (−0·005 to −0·002)	−0·001 (−0·003 to 0·001)	NS	NS	NS
Slope 2 statistically different from 0 p value	<0·0001	<0·0001	NS	··	··	··
Slope 1 *vs* slope 2 p value	0·013	<0·0001	0·0007	··	··	··

Data are estimates (95% CI), unless otherwise specified. 2-LTR=2-long terminal repeat. ART=antiretroviral therapy. NS=not significant.

* Measured in log_10_ HIV copies per mL of plasma.

† Measured in log_10_ ([HIV copies per 10⁶ CD4 cells] + 1).

**Table 3:** Models for the size of the inducible HIV reservoir

	Group estimates			Intergroup comparisons		
	Acute initiation of ART	Early initiation of ART	Deferred initiation of ART	Acute *vs* early p value	Acute *vs* deferred p value	Early *vs* deferred p value
**TILDA ex vivo** *						
Intercept	1·912 (1·529 to 2·296)	1·330 (1·046 to 1·613)	1·044 (0·806 to 1·283)	0·078	0·0009	NS
Slope	−0·179 (−0·237 to −0·121)	−0·102 (−0·145 to −0·058)	−0·081 (−0·115 to −0·046)	NS	0·022	NS
**TILDA after stimulation** *						
Change point, weeks	6	6	6	··	··	··
Intercept	2·441 (2·134 to 2·748)	2·031 (1·787 to 2·276)	1·573 (1·361 to 1·785)	NS	<0·0001	0·061
Slope 1	−0·266 (−0·311 to −0·221)	−0·170 (−0·204 to −0·136)	−0·143 (−0·170 to −0·115)	0·010	<0·0001	NS
Slope 2	−0·002 (−0·003 to 0·000)	−0·001 (−0·002 to 0·001)	0·001 (0·000 to 0·002)	NS	0·052	NS
Slope 2 statistically different from 0 p value	NS	NS	NS	··	··	··
Slope 1 *vs* slope 2 p value	<0·0001	<0·0001	<0·0001	··	··	··

Data are estimates (95% CI), unless otherwise specified. ART=antiretroviral therapy. TILDA=tat/rev induced limiting dilution assay. NS=not significant.

* Measured in log_10_ (frequency of CD4 cells with multiply-spliced RNA per million cells) + 1.

## Data Availability

All aggregated data are available either in the manuscript or appendix. Further requests might require ethical approval and should be discussed with the authors by contacting the corresponding author.

## Research in context

### Evidence before this study

It is well established that antiretroviral therapy (ART) initiation within the first few weeks or months of infection restricts the size of the HIV reservoir; inhibiting viral replication in the early stages of HIV infection limits establishment of a large pool of latently infected cells in the blood and tissues. However, a small pool of latently infected cells is always unavoidably established. Whether early ART initiation accelerates the decay of the latent HIV reservoir remains unclear. We searched PubMed on June 5, 2020, using the search terms “HIV”, “reservoir decay”, and “early ART”. We searched for articles published from database inception up to June 5, 2020, with no language restrictions. Even though we found several (n=6) studies that examined the effect of early ART initiation on the HIV reservoir kinetic in children, only a few (n=8) reports assessed the effect of early treatment initiation in adults. These studies often compared HIV DNA decay in people initiating ART 45–70 days after infection with participants starting ART during chronic infection. We did not identify any studies comparing the effect of early ART initiation on the decay of the inducible HIV reservoir over 4 years in people initiating ART within 30 days of infection. In addition, whether the benefits of starting ART within the first 2 months of being infected extend to individuals initiating therapy after 2 months of being infected remain unknown.

### Added value of this study

We measured the size of the HIV reservoir in 56 individuals who had been infected for less than 90 days, enrolled in the MERLIN clinical study in Peru. Participants were stratified on the basis of whether they initiated ART at 30 days or less (acute group), at 31–90 days (early group), or more than 24 weeks (deferred group) after the estimated date of detectable infection and we compared the size and rate of decay of the HIV reservoir among these groups. Longitudinal analysis over 4 years of ART revealed a biphasic decay of all HIV persistence markers, with a rapid initial decline followed by a slower decay in all participants. During the first phase, the half-lives of total and integrated HIV DNA and tat/rev induced limiting dilution assay measures were significantly shorter in the acute group compared with the early and deferred groups. During the second phase, HIV reservoir markers continued to decline only in participants in the acute group.

### Implications of all the available evidence

Our study suggests that initiation of ART during the early chronic phase of infection (ie, deferred initiation) is still beneficial when compared with infection of longer or unknown duration. These findings have important implications for HIV diagnosis strategies in high-risk populations, because they suggest that recurrent testing, and therefore early diagnosis and prompt ART initiation, could restrict the size of the HIV reservoir, including in individuals who have been infected for 2–3 months.
