# Supplementary material for: Long-term effects of early antiretroviral initiation on HIV reservoir markers: a longitudinal analysis of the MERLIN clinical study
Source: Lancet Microbe. Author manuscript; Available in PMC 2021 Nov 26. (PMC8622834; doi:10.1016/s2666-5247(21)00010-0)
Supplement: 1 [file NIHMS1757883-supplement-1.pdf]

THE LANCET
Microbe

Supplementary appendix

This appendix formed part of the original submission and has been peer reviewed. We post it as supplied by the authors.

Supplement to: Massanella M, Ignacio RAB, Lama JR, et al. Long-term effects of early antiretroviral initiation on HIV reservoir markers: a longitudinal analysis of the MERLIN clinical study. *Lancet Microbe* 2021; published online March 22. https://doi.org/10.1016/S2666-5247(21)00010-0.

Appendix

Long-term effects of early ART initiation on HIV reservoir markers

Marta Massanella, Rachel Bender Ignacio, Javier R. Lama, Amélie Pagliuzza, Sayan Dasgupta, Ricardo Alfaro, Jessica Rios, Carmela Ganoza, Delia Pinto-Santini, Trupti Gilada, Ann Duerr, Nicolas Chomont on behalf of the MERLIN study group.

The MERLIN study group

Fred Hutch
Ann Duerr
Sayan Dasgupta
Siavash Pasalar
Delia Pinto-Santini
Lili Peng
Carolyn Bain
Trupti Gilada
Jinru Tao

UdeM
Nicolas Chomont
Marta Massanella
Amelie Pagliuzza

UCSF
Joseph Mike McCune
Peter Hunt
Susan Lynch

Impacta
Javier R. Lama
Rosa Infante
Mey Leon
Javier Valencia
Carmela Ganoza
Luis Limo
Maria Mamani
Ricardo Alfaro
Maria Del Rosario
Josimar Vera
Pedro Gonzales
John Mac Rae
Helen Chapa
Jessica Rios
Karin Sosa

UW
Jim Mullins
Lisa Frenkel
Rachel Bender Ignacio

eMethods

Tat/rev Induced Limiting Dilution Assay (TILDA)

Productively infected cells and inducible HIV reservoir were measured by the Tat/rev Induced Limiting Dilution Assay (TILDA *ex vivo* and after stimulation, respectively). For samples obtained from before and during the first 8 weeks of ART (viremic samples), a fraction of the isolated CD4 T cells were distributed in limiting dilutions following CD4 T-cell enrichment to quantify the frequency of cells that spontaneously produce HIV multiply-spliced RNA (msRNA [tat/rev], TILDA *ex vivo*). In addition, a fraction of CD4 T cells from all samples was stimulated with 100ng/mL phorbol 12-myristate 13-acetate (PMA) and 1µg/mL ionomycin for 12 hours prior to TILDA measurement to assess the frequency of cells with inducible tat/rev RNA (stimulated TILDA). CD4 T cells (*ex vivo* or after stimulation) were stained to measure viability and activation using the following panel of antibodies: CD3-AF-700, CD4-APC, CD8-PerCP-cy5.5, CD69-PE-Cy7 (all from BD) and Aqua vivid Live-dead (Life Technologies). Only samples with >50% viable cells and >95% activated cells (as measured by CD69 expression) were included in the analysis. Following PCR amplification, positive wells at each dilution were counted and the maximum likelihood method was used to calculate the frequency of cells with inducible HIV msRNA (http://bioinf.wehi.edu.au/software/elda).

Longitudinal statistical analysis

The decays in viral loads and in all HIV reservoir measures were modeled from the time of ART initiation in each of the three groups until the end of

follow-up. The decay of each of these measures was analyzed on the $\log_{10}$ scale with a mixed-effects model to account for correlation between measurements from the same individual. The mixed effects model can account for individuals having different number of measurements and different measurement times. We used a piecewise linear function to model the decay, where the number of linear segments was fixed at 2 (two-phase segmentation model) for all HIV reservoir measures (except for TILDA *ex vivo*). For each HIV measure and each group (Acute, Early and Deferred), we built several models to determine the best change point of the two-phase segmentation model, which was selected based on minimization of the Akaike Information Criterion (AIC). The final model for each measure thus incorporated a known change point for each group, with different slopes on each side of the change point. It also included a fixed and a random effect for the intercept, but only fixed effects were assigned for the two slopes to avoid convergence issues. For TILDA *ex vivo*, the decay for this measure was modeled as a linear function.

eFigure 1

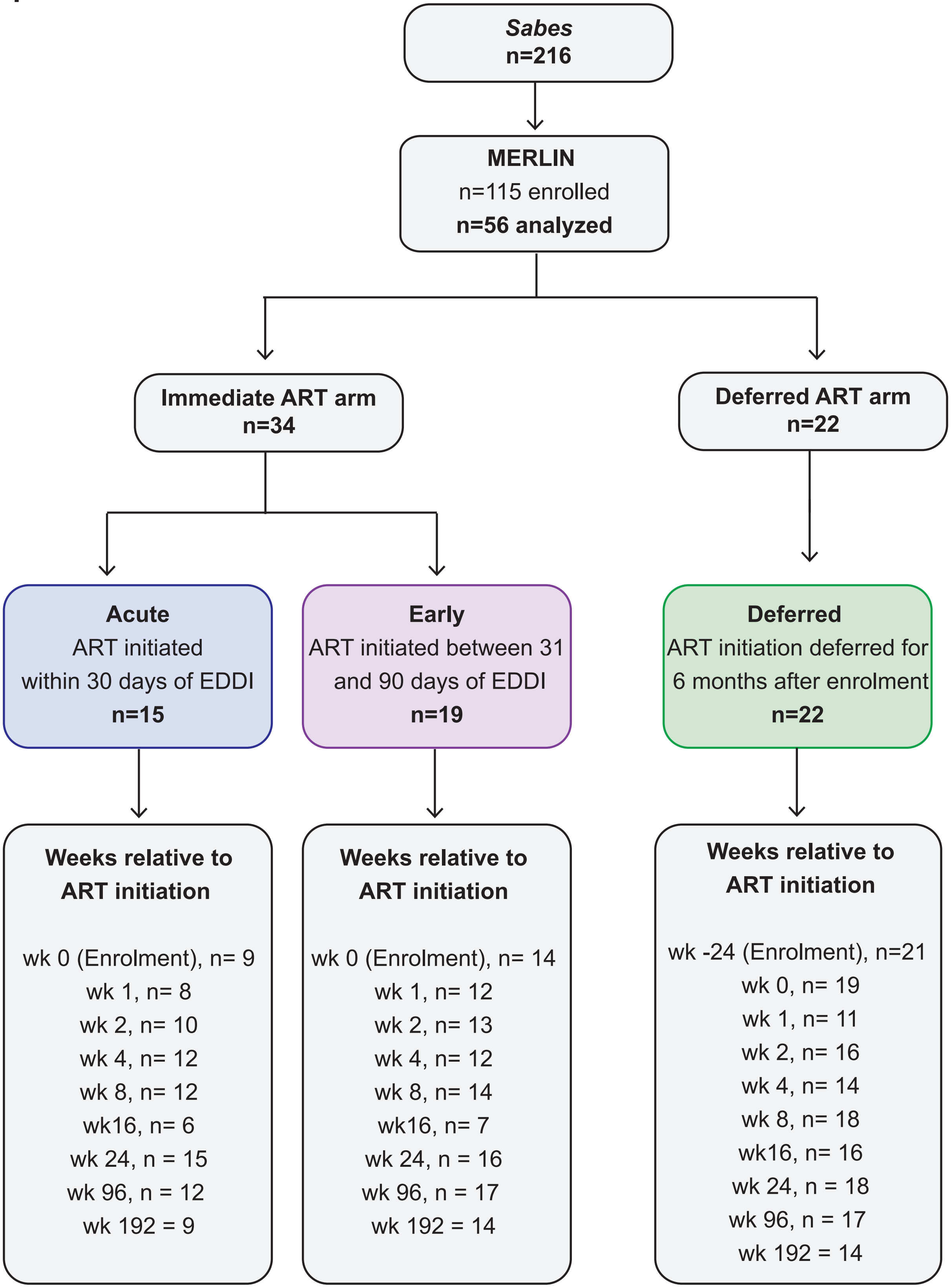

eFigure 1: Flow chart of the MERLIN study. Number of samples analyzed at each time point for the longitudinal analysis are indicated.

eFigure 2

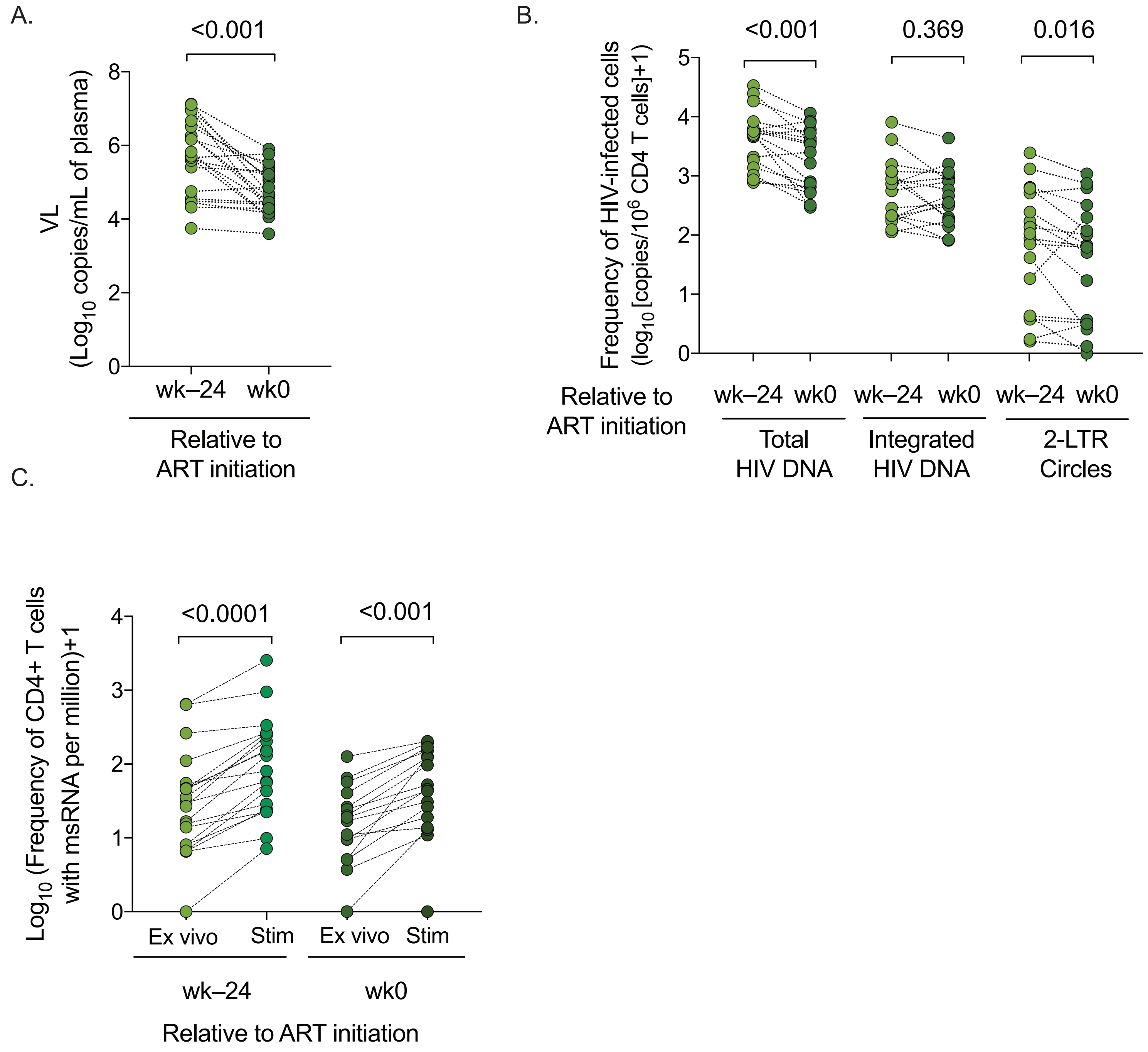

<u>eFigure 2:</u> Markers of HIV persistence at enrollment (week 0) and after 24 weeks in the deferred group. Samples from deferred group participants were collected at enrollment (week 0, light green) and at 24 weeks (week 24, dark green), on the day of ART initiation. Plasma viral load (n=21) (A), frequency of cells harboring total and integrated HIV DNA, and 2-LTR circles (n=18) (B), and frequency of cells producing msRNA spontaneously (TILDA ex vivo) or after 12h stimulation (inducible reservoir, n=20 and n=17 at week –24 and wk0 relative to ART initiation, respectively (C). P-values were obtained from Wilcoxon test for paired samples.

eFigure 3

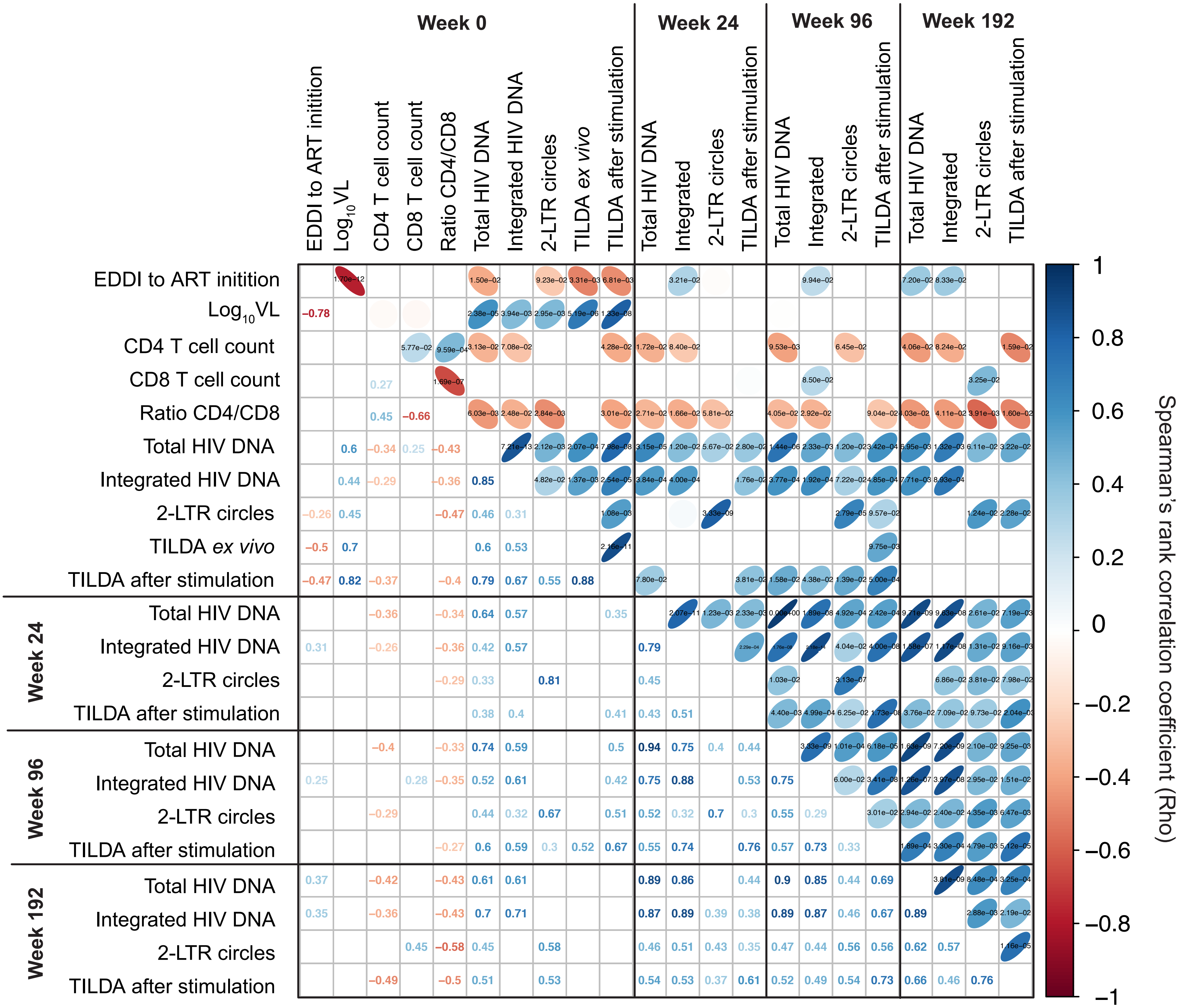

eFigure 3: Correlation matrix between markers of HIV persistence before treatment initiation and at different time points after ART initiation (wk24, 96 and 192). Rho values from Spearman correlation test are indicated in the bottom left part of the matrix and are reflected by the color of the ellipses (blue and red for positive and negative correlations, respectively). The shape of the ellipses reflects p-values (tighter ellipse reflects lower p-value), which are also indicated in the upper part of the matrix. Only correlations with a p-value <0.1 are shown.

eTable 1: Characteristics of participants in the deferred group at enrollment (Wk –24) and 6 months after (wk0), before ART initiation

Weeks from diagnosis	Deferred groupd (n=22)		Wilcoxon test
	wk0	wk24	*p-value*
Time from EDDI to Analysis (days), median [IQR]	38 [27-57]	206 [194-223]	***<0.001***
CD4 T cell counts, cells/µl, median [IQR]	419 [299-547]	402 [318-573]	*ns*
CD4, %, median [IQR]	24 [19-32]	24 [18-28]	*ns*
CD8 T cell counts, cells/µl, median [IQR]	838 [564-1057]	735 [562-925]	*ns*
CD8, %, median [IQR]	43 [36-59]	41 [37-48]	*ns*
Ratio CD4/CD8, median [IQR]	0.50 [0.35-0.86]	0.62 [0.34-0.68]	*ns*
log_{10}VL (copies/mL), median [IQR]	5.71 [4.65-6.59]	4.59 [4.25-5.26]	***<0.001***
Total HIV DNA, log_{10}(x+1), HIV copies/10^6 CD4 T cells, median [IQR]	3.71 [3.24-3.83]	3.47 [2.79-3.74]	***<0.001***
Integrated HIV DNA, log_{10}(x+1), HIV copies/10^6 CD4 T cells, median [IQR]	2.81 [2.30-3.06]	2.61 [2.25-2.99]	*ns*
2-LTR circles, log_{10}(x+1), HIV copies/10^6 CD4 T cells, median [IQR]	1.99 [0.63-2.73]	1.8 [0.51-2.35]	***0.016***
TILDA *ex vivo*, log_{10}(x+1), HIV copies/10^6 CD4 T cells, median [IQR]	1.45 [0.91-1.72]	1.23 [0.71-1.51]	ns
TILDA after stimulation, log_{10}(x+1), HIV copies/10^6 CD4 T cells, median [IQR]	2.01 [1.41-2.41]	1.66 [1.21-2.14]	ns
